# Deferoxamine Supplementation Abolished Iron-Related Toxicity of *Ilex paraguariensis* Extract: Behavioral and Biochemical Evaluation in Adult Zebrafish (*Danio rerio*)

**DOI:** 10.3390/antiox11081507

**Published:** 2022-07-31

**Authors:** Wagner Antonio Tamagno, Carla Alves, Diego Tessaro, Nathália Tafarel Sutorillo, Wallace Santin, Leonardo José Gil Barcellos

**Affiliations:** 1 Biochemistry Profª Drª Rosilene Rodrigues Kaizer Laboratory of the Federal Institute of Education, Science, and Technology of Rio Grande do Sul, Sertão Campus, Sertão 99170-000, RS, Brazil; wagner.tamagno@sertao.ifrs.edu.br (W.A.T.); carla.alves@sertao.ifrs.edu.br (C.A.); 2017004368@aluno.sertao.ifrs.edu.br (D.T.); 343188@aluno.sertao.ifrs.edu.br (N.T.S.); 231193@aluno.sertao.ifrs.edu.br (W.S.); 2 Graduate Program in Pharmacology, Universidade Federal de Santa Maria (UFSM), Santa Maria 97105–900, RS, Brazil; 3 Graduate Program in Bioexperimentation, Universidade de Passo Fundo (UPF), Passo Fundo 99052–900, RS, Brazil; 4 Graduate Program in Environmental Science and Technology, Federal University of Fronteira Sul (UFFS), Erechim Campus, Erechim 99700-970, RS, Brazil

**Keywords:** antioxidant, herb mate, deferoxamine, oxidative stress, microencapsulation, behavior

## Abstract

*Ilex paraguariensis* (Herb mate) is a native plant from South America, widely consumed through the infusion of dried leaves. The presence of antioxidant properties in herb mate may be relevant and contribute to evaluating the effect of its compounds against oxidative stress, which could cause neurodegenerative diseases. Despite having health benefits, there are reports of the presence of heavy metals in extracts obtained from the infusion. One of these metals is iron (Fe), found in large amounts in herb mate. To reverse the cumulative effects of metals and Fe in the body, the use of Deferoxamine (Dfx) is indicated, being a potent chelator of Fe. In this work, we aimed to evaluate the antioxidant potential of the micro-encapsulated extract of *I. paraguariensis* (MEIP) supplemented with Dfx on zebrafish behavior and biochemical biomarkers. To evaluate the effect *per se* and the supplementation, four groups were established: the first group was the control (water); the second, fish treated with MEIP; the third group was formed of fish treated with Dfx; while the fourth group was treated with both MEIP and Dfx. When applied alone, Dfx presents an anxiogenic-like pattern on zebrafish (*Danio rerio*), while the MEIP shows an anxiolytic-like behavior. The antioxidant enzymes are re-modulated close to control when the MEIP + Dfx is applied. The cholinergic system shows an activation of the signaling, as well as the heme radical group formation, which is not affected by the Dfx-chelating effect. Thus, the supplementation of MEIP with Dfx is important to transform this extract into one that is safer and healthier for human consumption.

## 1. Introduction

Herb mate (*Ilex paraguariensis*, St. Hill) is a native species from South America and its natural range is restricted to Brazil, Paraguay, and Argentina [1]. This plant is used to prepare, through the infusion of its dried and ground leaves, the mate tea, called “chimarrão” in southern South America. The ingestion of herb mate extracts has the potential to increase the antioxidants in the system, reducing free radical attacks [2]. This plant has great antioxidant potential due to the presence of polyphenols, which constitute about 11% of the dry matter weight [3]. Its antioxidant properties are associated with the high content of phenolic compounds, mainly chlorogenic acid and its derivatives of (3,4-di-O-caffeoylquinic), (3,5-di-O-caffeoylquinic), and (4-acid-S-dicapheoylquinic 5-caffeic-acid), as well as the presence of flavonoids, such as quercetin, rutin, kaempferol, and luteolin [4].

Oxidative stress has its damage minimized by the non-enzymatic or enzymatic antioxidant defense system; its evaluation is largely used by the activity of the enzymes superoxide dismutase (SOD), catalase (CAT), and also glutathione-S-transferase (GST) [5]. Although it receives attention due to its high content of bioactive compounds, herb mate can have its effects reduced by external and internal factors, due to the high sensitivity of these compounds to temperature, oxidation, pH, light [6,7], climate, soil, and cultivation conditions, among others. In this context, there has been progress in recent studies to improve the composition of the extract of herb mate, with the use of concentration technologies, due to the great diversity of phytochemicals with functional properties, such as phenolic compounds, alkaloids, flavonoids, and terpenoids. The spray-dryer technique is one example of extract preparation that uses a pressurized extract with gum under a high temperature for a short period, aiming to dry this by forming small spheres to conserve the extract [8,9,10]. This advance allowed for its application in the production of medicines and cosmetics, as well as in the development of new products and ingredients for food supplementation.

The presence of high concentrations of iron as well as other heavy metals in the soil can be harmful to the biota [11]. The plants can absorb the heavy metals present in the soil and mobilize them to the shoot as a defense mechanism against toxicity. In plants, after being absorbed, the metals react with the cell walls and can harm the plant; in photosynthetic systems, they interact harmfully with photosystems I and II, as well as in the biochemical phase [12]. Due to high toxicity, as a defense mechanism, since plants cannot eliminate these compounds, the storage and rearrangement of the heavy metals in plastids occur. In the extraction of plants for teas, the heavy metals stored in the vesicles are collected together [13]. In the processes of the extraction of the phytochemical compounds, such as in a boiling decoction, these metals can be more efficiently extracted and toxic to those who consume them [14]. In a work carried out by De Bortoli et al. [6], it was observed that a chimarrão, a similar decoction, of *Ilex paraguariensis* at 85 °C resulted in the extraction of high concentrations of iron, aluminum, and lead and that these metals were toxic in an in vivo assay. This metal accumulation occurs because the herb mate grows naturally in soils with an acidic pH, which promotes an increase in the bioavailability of the ions, which are easily solubilized in the form of basic salts, such as Fe [15].

To reverse this cumulative effect of Fe, and other metals, the World Health Organization recommends the use of Deferoxamine (Dfx), commonly used for the treatment of various hematological diseases [16]. Dfx has a good safety profile and is a potent iron chelator, where it has been studied in cardiovascular events and sepsis [17].

The use of model organisms is a tool that allows for the evaluation of the effect of the consumption of substances with antioxidant potential, such as the microencapsulated extract of *I. paraguariensis* (MEIP) and Dfx, on behavioral and biochemical parameters. Zebrafish (*Danio rerio*) are a good choice as a model organism, due to their rapid reproductive capacity [18], their sequenced genome [19], and their defined developmental process [20,21,22]. These fish have the potential to evaluate the toxicity of compounds in experiments. Because of the high levels of consumption of mate tea and its heavy metal extraction, here we evaluated the effect of supplementing microencapsulated extract of *Ilex paraguariensis* (MEIP) with a potent metal chelator Dfx on the behavioral and biochemical biomarkers of zebrafish, aiming to understand the process of the chelation of heavy metals present in herb mate, to make the tea safer and healthier for human consumption.

## 2. Materials and Methods

### 2.1. Study Strategy

Given that, we carried out the extraction of the tea from *Ilex paraguariensis* at 85 °C and microencapsulated the extract, using maltodextrin as the encapsulating agent (creating the MEIP). Dfx was used as the heavy metal-chelator at the concentration of 180 mg·L^−1^. To evaluate the effect per se and the supplementation, four groups were established: the first group was the control (freshwater); the second group was the fish treated with MEIP (100 mg·L^−1^); the third group was formed by the fish treated with Dfx (180 mg·L^−1^); while the fourth group was treated with both MEIP (100 mg·L^−1^) and Dfx (180 mg·L^−1^). The Dfx was obtained commercially from Sigma-Aldrich (CAS number 70-51-9, Merck KGaA, Darmstadt, Germany). The effect was evaluated in the zebrafish exposed to the Dfx and MEIP in the aquarium water once a day for 7 days and then the behavioral and biochemical analyses were completed, as described in the following sections.

### 2.2. Ilex paraguariensis Extract Preparation, Microencapsulation, and Efficiency of Encapsulation Rate

The herb mate tea chosen for the MEIP preparation in this work comes from a company in the northern region of the state of Rio Grande do Sul—Brazil and its industrialization process [7].

To obtain the liquid extract for microencapsulation, a proportion of the processed mate tea and water of 1:3 (*w*/*v*) was used and kept in decoction for 30 min at 85 °C [7], similar to the chimarrão preparation. Then, it was filtered through filter paper (45 µM) at room temperature and transferred to Falcon tubes protected from the light and kept at −80 °C until the spray-dryer process. The microencapsulation process was followed, according to the procedure described by Tamagno et al. [10], dried in a drying chamber (LM-MSD 0.5), using inlet air at 120 °C and outlet air at 76 °C, with a flow rate of 4.5 m^3^·min^−1^, and under 4.5 Kgf·cm^−2^ pressure. The flow rate of the extract peristaltic pump was 1.0 L·h^−1^ and that of the compressed air through the nozzle was 35 L·min^−1^. Maltodextrin (16:5–19:5 dextrose equivalent, Sigma-Aldrich, Merck KGaA, Darmstadt, Germany) was used as an encapsulating agent at a proportion of 2:1 (m:m).

The efficiency of the encapsulation was determined in the MEIP, according to Gómez-Mascaraque et al. [20], and Ghorbanzade et al. [21], with some adaptations as described by Tamagno et al. [10]. In addition, the morphology of the particle was observed using conventional electronic scanning microscopy.

### 2.3. Fish

Adult (200 fish), mixed-sex zebrafish (*Danio rerio*—wild type), 3 to 4 cm were used in this study. They were acclimated in a density of 2 fish.L^−1^ for 15 days before being exposed. The photoperiod was time-controlled with 12 h light: 12 h dark. The water temperature was kept at 28 °C with pH = 7.0, dissolved oxygen at 6.5 ± 0.4 mg·L^−1^, total ammonia at <0.01 mg·L^−1^, total hardness at 50 ± 5 mg·L^−1^, and alkalinity at 40 ± 3 mg·L^−1^ CaCO_3_. They were fed twice a day with flaked food (Alcon Basic, Alcon, Camboriú, Brazil). The current experiments were approved by the Ethics Committee on the Use of Animals of the Federal Institute of Rio Grande do Sul (CEUA/IFRS), according to protocol No. 7849300320.

### 2.4. Standards

Acetylthiocholine iodide, bovine albumin, Coomassie blue (G25), hydrogen peroxide, acetonitrile, and 5,5-dithiobis-2-nitrobenzoic-acid (DTNB) were purchased from Sigma-Aldrich Corp. (St. Louis, MO, USA). The purity of the standards was at least 95%, which is the level of high-performance liquid chromatography (HPLC).

### 2.5. Behavioral Analyses

The behavioral analyses were video recorded, using a Logitech C525 and analyzed with ANYmaze^®^ 7.1 tracking software (Stoelting Co., 620 Wheat Lane Wood Dale, IL 60191 USA). For the two behavioral tests, we used a glass aquarium (15 × 25 × 5 cm (w × h × d)) filled with 7 L of water, with the same characteristics as the experimental aquarium. Two behavioral tests were conducted in triplicate. After the fish were euthanized, the homogenate was prepared for the biochemical tests. Fourteen fish were used for the Novel Tank Test (NTT), which was completed according to a protocol previously used by Dametto et al. [22]. The fish were kept in aquaria and recorded for 6 min. The aquaria were divided into three horizontal segments, and the parameters of time in the top zone(s) were evaluated, the time in the bottom zone(s), the distance traveled(m), and the latency to the top(s). The social preference test (SPT) of 24 fish per group was evaluated, as described by Gerlai [23]. In this, the fish were kept in a tank where a group of conspecifics was placed on one side, and on the other side, an empty aquarium was kept. The fish were individually left for acclimatization for 30 s, with an opaque barrier between both of the sides. After 30 s, the barrier was removed and the social behavior was recorded for 2 min. The test aquarium was divided into three vertical segments and the parameters of time in the closer segment(s), number of crossings, and time in the empty segment(s) were evaluated.

### 2.6. Biochemical Analyzes

The extract for CAT, GST, SOD, TBARS, ALAD, and AChE in the brains of the fish was prepared, pooling three brains and homogenizing them in an ice bath 60 vol of TFK buffer (11 mM, pH 7.5) in a Potter-homogenizer for 60 s. The H_2_O_2_ converting rate by the CAT activity was determined, as described by Goth and Johansson and Borg [24,25]. The GST activity was determined, as described by Habig et al. and Habig and Jakoby [26,27]. The superoxide ion (O_2_^−^) dismutation and H_2_O_2_ formation rate were determined by the SOD activity, and were determined as described by Misra and Fridovich [28]. The MDA levels were evaluated by TBARS, as described by de Quadros Camargo et al. [29]. The conversion of acetylcholine to acetate and choline by the AChE activity was determined as described by Ellman et al. and by Kaizer et al. [30,31]. The porphobilinogen formation was determined by the ALAD (delta-aminolevulinic acid dehydratase) activity was determined according to Sassa [32].

To determine the lipid peroxidation by ferrous oxidation-xylenol (FOX) in brain tissue, two brains were homogenized in a solution with methanol (100%) and butylated-hydroxy-toluene (BHT; 0.01%). The extract was vortexed for 1 min and centrifuged at 1000× *g* for 10 min at 4 °C. The supernatant was collected to be used on the FOX test that was performed, according to Moore and Roberts [33].

The whole body was entirely crushed and extracted for cortisol determination, and then using ELISA assay (Cortisol ELISA kit; Diagnostics Biochem Canada. 384 Neptune Crescent, London, Ontario, CA) [34,35].

### 2.7. Statistics

The data of the three replicates were analyzed using two-way ANOVA (with the presence (+) or absence (−) of MEIP and Dfx as independent factors); followed by Tukey’s post-hoc test, depending on the normality of the data (assessed by the Kolmogorov–Smirnov test). The graphs were constructed using the Graph Pad Prism 8.0.1 Software (GraphPad Software 2365 Northside Dr. Suite 560 San Diego, CA 92108). Each graph is represented by the mean ± standard error and asterisks represent statistical difference among the groups.

## 3. Results

All of the statistic details, such as as F and the *p* values of all of the comparisons completed, are shown in Table 1.

### 3.1. Microencapsulation Evaluation by Scanning Electron Microscopy and Efficiency Determination

**Figure 1:**
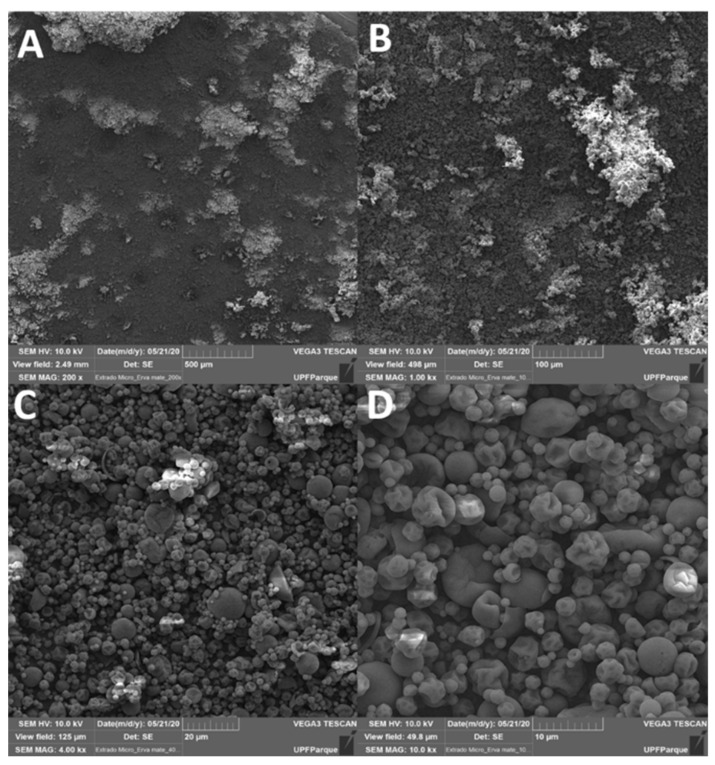
Scanning electron microscope (Phenom XL, Thermo scientific) in MEIP. (**A**) 200×; (**B**) 1000×; (**C**) 4000×; (**D**) 10000× of magnification.

After the efficiency of the encapsulation, was observed that 99.57% of the MEIP was completely encapsulated into the microcapsules of maltodextrin. In addition, the particle morphology by the spray-dryer (Figure 1), presented a globose shape of an irregular size with a widely wrinkled surface, highlighting that they were not filled with the extract. In addition, there were no extracts visible on the outside of the particles, and there were no cracks on the wall of the capsule, indicating that the process and encapsulation were efficient.

### 3.2. Behavior

#### 3.2.1. Novel Tank Test (NTT)

**Figure 2:**
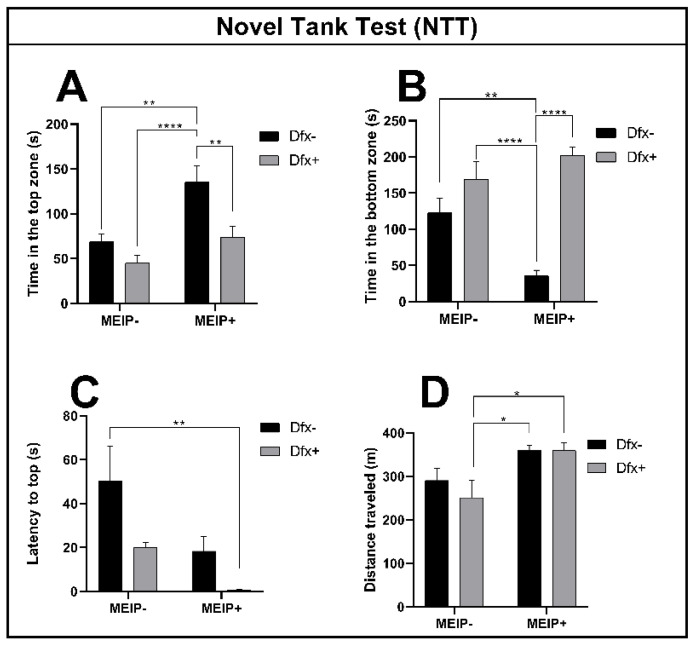
Novel Tank Test (NTT) in Zebrafish. Representation of the time in the top zone (**A**); time in the bottom zone (**B**); latency to top zone (**C**); distance traveled (**D**) of fish exposed to Dfx supplemented MEIP. Data are expressed by the mean ± SEM analyzed by ANOVA two-way with Tukey’s post-hoc test. * *p* < 0.05, ** *p* < 0.01 and **** *p* < 0.0001.

The fish exposed to the MEIP+ (Figure 2A), remained on top of the aquarium for a longer time in comparison to the control, Dfx+, and MEIP + Dfx. The results were proportional to the time the fish spent at the bottom of the aquarium (Figure 2B) in comparison to the control, Dfx+, and MEIP + Dfx. The latency time (Figure 2C) for entry to the top was decreased in the group treated with MEIP + Dfx in comparison to the control. The distance traveled (Figure 2D) was increased in comparison to Dfx+ in the groups MEIP+ and MEIP + Dfx. When the fish remain more at the top of the aquarium, it is related to the fact that they are less anxious and are exploring the new environment. On the other hand, when the fish remain more at the bottom of the aquarium, it is related to anxiety as well as to fear.

#### 3.2.2. Social Preference Test (SPT)

**Figure 3:**
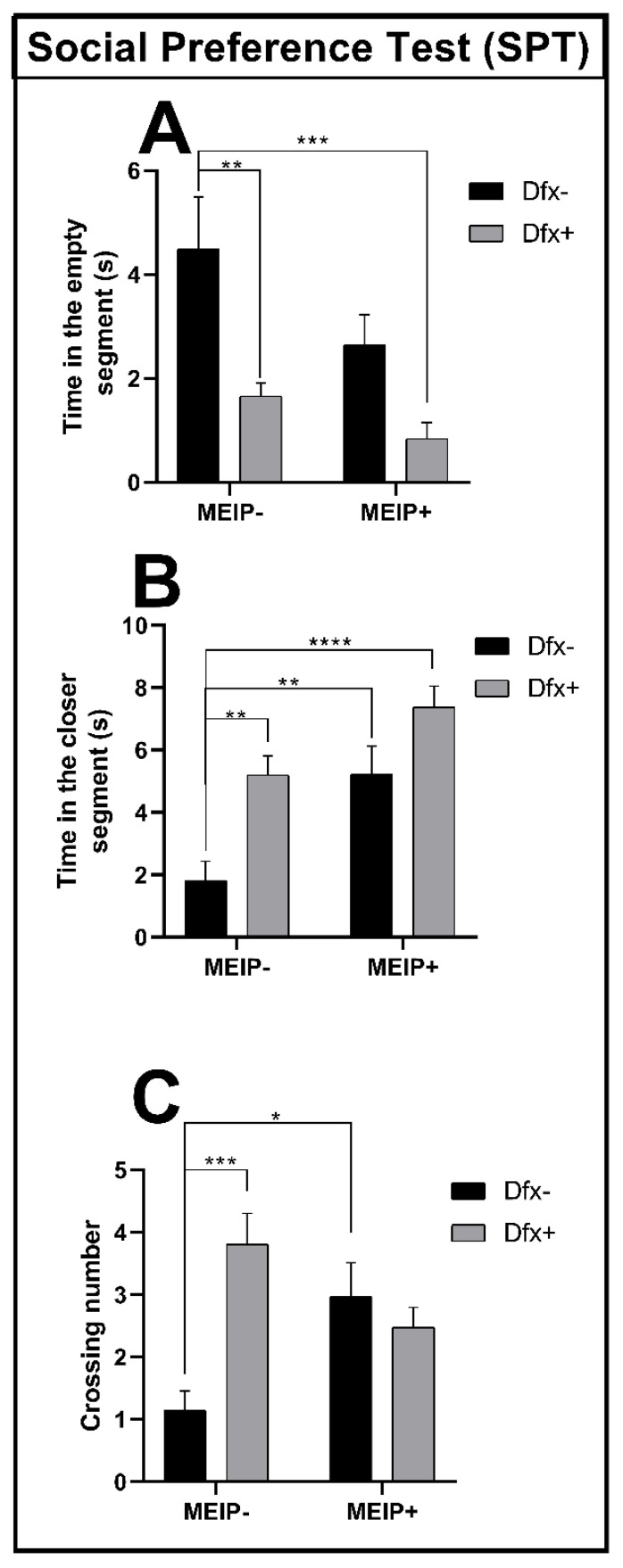
Social Preference Test (SPT) in Zebrafish. Representation of the time in the empty segment (**A**); time in the closer segment (**B**); and number of line crossings (**C**) of fish exposed to Dfx supplemented MEIP. Data are expressed by the mean ± SEM analyzed by ANOVA two-way with Tukey’s post-hoc test. * *p* < 0.05, ** *p* < 0.01, *** *p* < 0.001, and **** *p* < 0.0001.

In the social preference test (Figure 3A), the fish treated with Dfx+ and MEIP + Dfx remained for a shorter time in the segment close to the conspecifics in comparison to the control group. The results were proportional to the time that the fish spent far from the conspecific zone (Figure 3B) showing that the time spent by the Dfx+, MEIP+, and MEIP + Dfx groups was increased in comparison to control. The crossing number (Figure 3C) was increased in the groups Dfx+ and MEIP+ in comparison to the control. The social preference test evaluates the social interaction of the fish with the conspecifics, and natural behavior is linked to a strong interaction between the fish and the conspecifics.

### 3.3. Biochemical

#### 3.3.1. ALAD Activity

**Figure 4:**
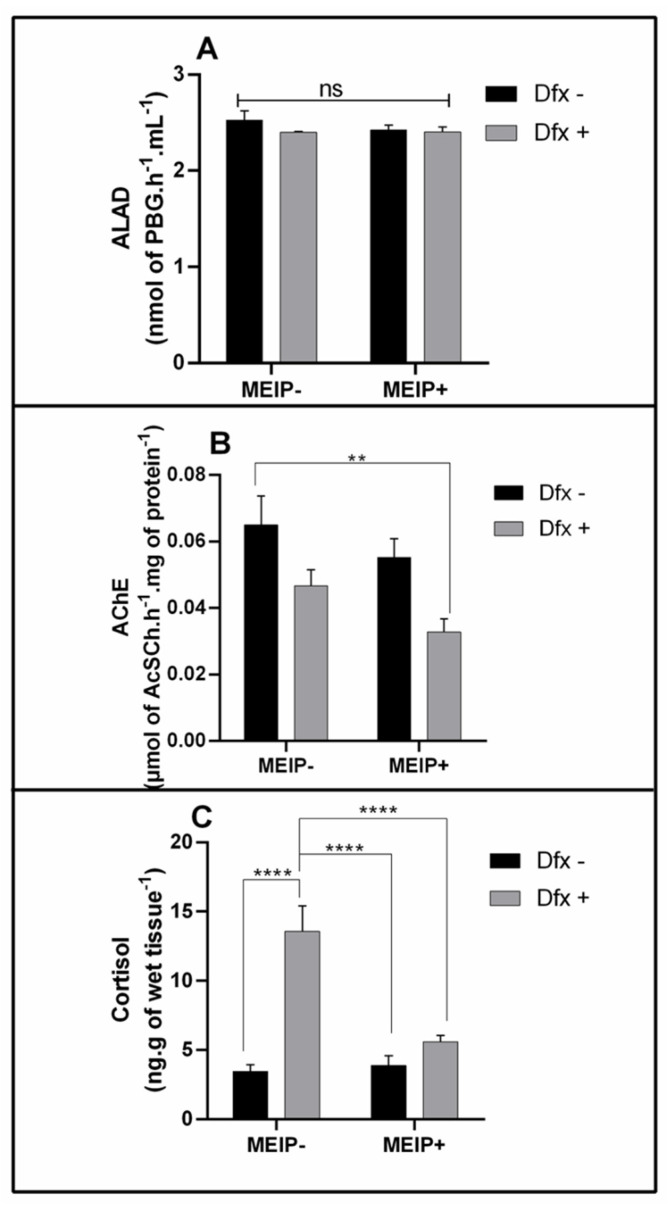
Delta-aminolevulinic acid dehydratase activity (ALAD) on Zebrafish brain (**A**); Acetylcholinesterase activity (AChE) on Zebrafish brain (**B**); and cortisol levels on Zebrafish whole body (**C**) exposed to Dfx-supplemented MEIP. Data are expressed by the mean ± SEM analyzed by ANOVA two-way with Tukey’s post-hoc test. ** *p* < 0.1 and **** *p* < 0.001, and ns = no significant.

The ALAD activity (Figure 4A) was not changed in any group treated with the MEIP+ and/or Dfx, as well as in its mixtures.

#### 3.3.2. AChE Activity

The AChE activity (Figure 4B) was decreased in the group treated with the MEIP + Dfx in comparison to the control group.

#### 3.3.3. Cortisol Levels

Cortisol levels (Figure 4C) increased in the group treated with Dfx+ in comparison to the MEIP + Dfx, MEIP+ and control groups.

#### 3.3.4. Antioxidant Status

##### Antioxidant Enzymes

**Figure 5:**
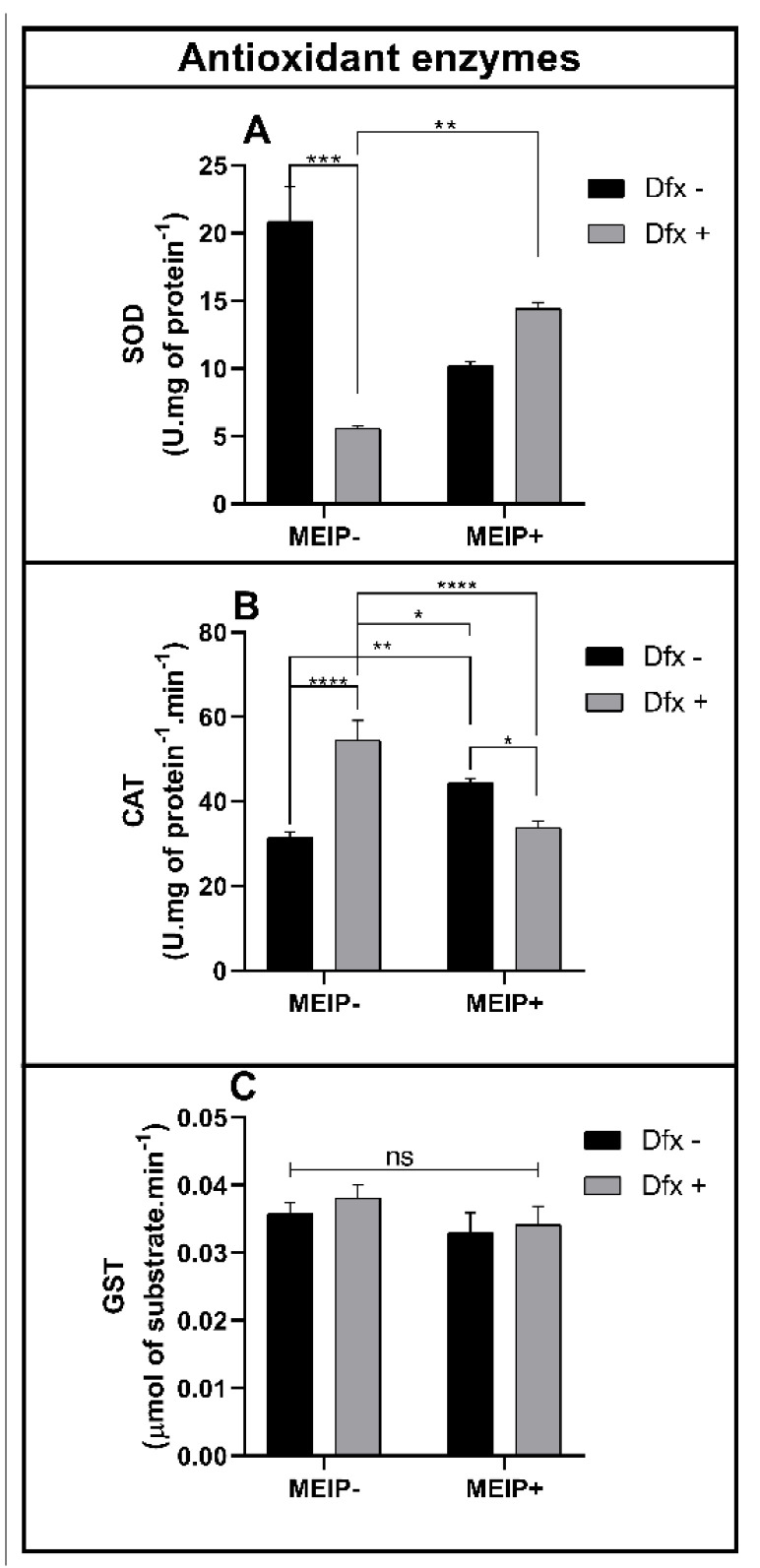
Superoxide dismutase (SOD) activity (**A**); catalase (CAT) activity (**B**); glutathione-s-transferase activity (**C**) on zebrafish brain exposed to Dfx supplemented MEIP. Data are expressed by the mean ± SEM analyzed by ANOVA two-way with Tukey’s post-hoc test. * *p* < 0.5, ** *p* < 0.1, *** *p* < 0.01; and **** *p* < 0.001, and ns = no significant.

The enzyme SOD (Figure 5A) was decreased in the group treated with the Dfx+ in comparison to the control and MEIP + Dfx. 

The CAT activity (Figure 5B) was increased in the group Dfx+ in comparison to the control, MEIP + Dfx, and MEIP. The group treated with the MEIP + Dfx was increased in comparison to the control. The group treated with Herb+ was increased in comparison to the MEIP + Dfx.

The GST activity (Figure 5C) was not changed in any of the groups treated with the MEIP and/or Dfx as well as its mixtures.

##### Lipid Peroxidation

**Figure 6:**
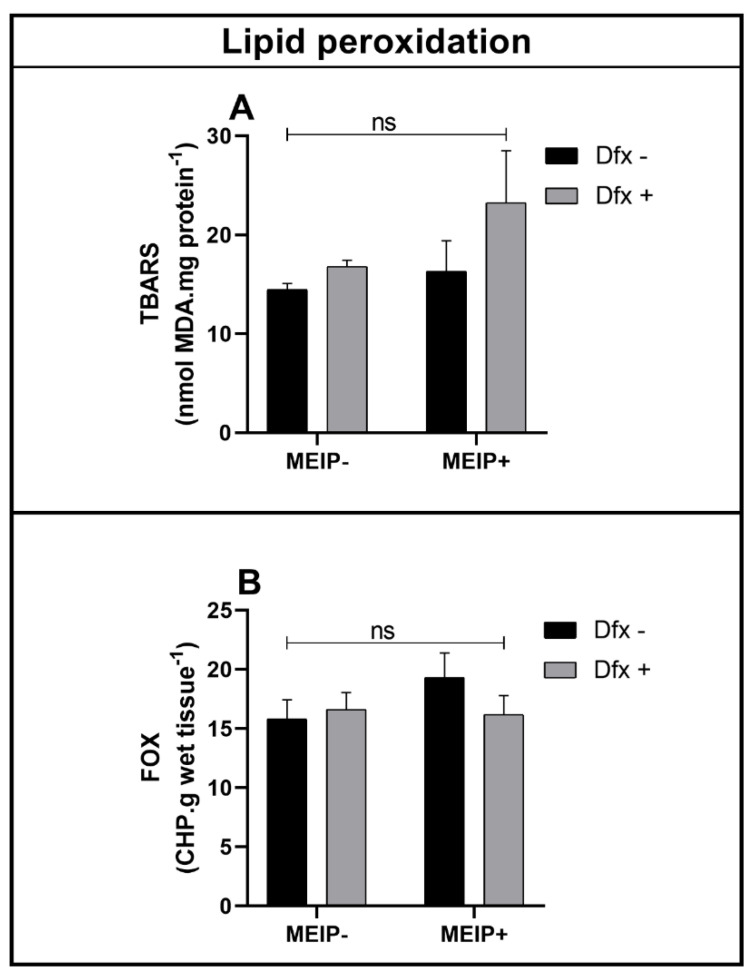
Lipid peroxidation by TBARS (**A**); FOX (**B**) on zebrafish brain exposed to Dfx supplemented of MEIP. Data are expressed by the mean ± SEM analyzed by ANOVA two-way with Tukey’s post-hoc test; ns = no significant.

Lipid peroxidation in the brain, even that accessed by TBARS (Figure 6A) and by FOX (Figure 6B), was not significant in any of the groups treated with the MEIP and/or Dfx, as well as its mixtures.

## 4. Discussion

Here, we show that the MEIP plays a role as an anxiolytic compound on zebrafish behavior, decreasing the cortisol levels and increasing the CAT activity. In addition, Dfx acts as an anxiolytic compound, increasing the time that the fish spend in the upper zone of the aquaria. Dfx also increases the cortisol levels. In the MEIP + Dfx treatments, the isolated effects of the MEIP and Dfx were abolished, and the fish maintained their natural behavioral responses to the tasks performed (NTT and SPT), similar to the control. The observed behavioral changes can be linked to the cortisol levels and AChE activity, suggesting the positive effect of using the MEIP + Dfx to minimize the effects of the Herb mate and Dfx used alone.

When evaluating the particles of the MEIP by scanning electronic microscopy, it is possible to verify that this process and protocol were effective in promoting a high efficiency of microencapsulation. The globose size of the particles, as well as the non-observed extract residues in the external structure of the particles, and the uniformity of the particles, indicated the encapsulation efficiency [10]. A possible limitation of our experimental design is that we did not confirm if the particles of encapsulation were loaded with the extract; we only observed the morphological structure and compared it with the encapsulation efficiency, allowing us to suggest that the encapsulation was efficient.

All of these results show a relation to the antioxidant potential of *Ilex paraguariensis,* which is widely described in the literature [2,3,36,37,38,39], even after the microencapsulation process [40,41,42]. In addition, the efficiency of the encapsulation process of *Ilex paraguariensis* is considered stable with the wall product used (maltodextrin gum Arabic). According to Fenoglio et al. [40], the high efficiency of the MEIP is attributed to the fact that maltodextrin is a material that tends to form spheres that will retain the extract during the homogenization process before the drying process. Furthermore, the maltodextrin can protect the extracts from the high temperatures during the process of spray-drying. The high temperatures and short process time may have increased the mass and heat transfer, thus achieving a better encapsulation yield [43].

The supplemented MEIP with Dfx has two objectives, first being its performance as a chelating compound of heavy metals and second, due to its performance as a non-enzymatic antioxidant. Dfx is considered to be a chelator of iron and aluminum, as it forms insoluble complexes, preventing them from entering chemical reactions [44]. Dfx toxicity and its potential for chelating were tested in the zebrafish embryos: it was not toxic in fish at concentrations ranging from 0 to 1000 µM [45]. In the human body, the Dfx chelates the free iron in serum, ferritin, and hemosiderin, not removing iron from the transferrin, hemoglobin, or cytochromes. Since herb mate extracts contain high concentrations of aluminum and iron [46], and because of the neurotoxic role of these compounds in the nervous system [47], the use of compounds that decrease the bioavailability of these toxic metals and inhibit neurotoxicity [48] is very important. Finally, Dfx can be used as an antioxidant, since its combination with natural antioxidant compounds can even potentiate its effect [49].

In the behavioral biomarkers evaluated, we observed that the MEIP plays an anxiolytic role, due to the increased time in the top zone in the NTT task. When the MEIP is applied with Dfx, the natural behavior is reestablished. The NTT test determines the anxious-like pattern of fish, as well as the exploratory and locomotor behavior. In a new environment, the expected behavior of the fish is to start in the bottom zone to avoid unnecessary dangers, and gradually the fish explore the new environment [50]. In the SPT test, the social pattern of the fish is evaluated. In our study, the fish became more sociable when the MEIP and Dfx were applied together. Zebrafish are a highly social species because they remain closer to their conspecifics; the social interaction among the individuals in the same species is very important to reproduction, predatory protection, and species perpetuation.

We observed that when Dfx and MEIP are applied together, the rate of AChE activity is decreased. AChE activity is related to the cholinergic signaling in the nervous system. This enzyme removes the neurotransmitter acetylcholine (ACh) from the synaptic cleft by the hydrolyzation of ACh to choline and acetate closing the calcium channels [31]. In this way, in our study, the ACh remained for a longer time in the synaptic cleft and the nervous system was activated. The activity was decreased and indicated that an activation of the nervous system had occurred. In neural human diseases, the AChE reduction is the most important effect for the treatment of diseases, such as Parkinson’s and Alzheimer’s [7].

No alteration was observed in the ALAD activity in any one group of the treatments, suggesting that there is no toxicological effect on heme formation. ALAD activity is related to the radical heme group formation that is very important in oxygen transport due to the hemoglobin [51]. The heme group is formatted by an iron-metallic center, and it can particularly interact with Dfx. 

In our work, we show that Dfx applied alone reduced the SOD activity. It may be due to the chelating effect of Dfx that reacts with heavy metals, especially with Cu, forming insoluble complexes and reducing the enzyme activity. When Dfx is applied with MEIP, the SOD activity is re-established to normal levels. This observation indicates that the Dfx content in the extract is already interacting with other metal compounds from the extract. This result shows that the isolated application of Dfx acts as a metallic chelator in the body, since it reduced the bioavailability of metallic cofactors, responsible for the maintenance of SOD. On the other hand, when applied in supplementation, it interacts before with the metals in the extract, thus reducing its deleterious effect on the body, since after the application of the MEIP with Dfx, the SOD levels were re-established. The antioxidant system is very important in controlling the reactive oxygen species and avoiding oxidative stress [52]. The first enzyme of the antioxidant line of defense is the superoxide dismutase (SOD). SOD works by reducing the superoxide radical (O_2_^−^), which is extremely reactive to hydrogen peroxide (H_2_O_2_), using a metallic cofactor such as copper (Cu), zinc (Zn), or manganese (Mn) [53]. The bioavailability of these cofactors directly affects enzyme activity. Such cofactors may differ according to the cellular compartments of enzyme action. The SOD can be found in two forms: in the cytoplasm, it is dependent on copper and zinc (SOD-Cu/Zn), while in the mitochondria, it requires manganese as a cofactor (SOD-Mn) [54].

Here we show that the catalase (CAT) activity was increased in the group treated with Dfx, which indicates that there was an increase in the amount of H_2_O_2_ formed. Unlike the SOD, the enzyme CAT does not have an enzymatic cofactor, so it cannot be affected by the bioavailability of metals chelated by Dfx. CAT works by reducing H_2_O_2_ to water (H_2_O) and oxygen (O_2_) [55]. In the antioxidant defense line, the CAT reacts with the H_2_O_2_ generated by the SOD [56]. Since the SOD activity in the Dfx group was reduced, the amount of H_2_O_2_ formed was also reduced. So, the explanation for the increase in H_2_O_2_ may be related to the blocking of the Fenton reaction (ferric or/and cuprous) by Dfx. The Fenton reaction uses a Fe or Cu ion to interact with H_2_O_2_, to form the hydroxyl radical (OH^−^) [57]. Due to the chelating effect of Dfx in the organism, the Fenton reaction did not occur and the amount of H_2_O_2_ was increased, thus increasing the activity of CAT. Finally, when the administration of the MEIP supplemented with Dfx is observed, once again the levels are reestablished, indicating that the chelating effect of Dfx has already occurred with the extract before reaching the body [58].

The enzyme Glutathione-S-transferase was not changed in this study, indicating that no toxic compound was generated and needed to be bio-transformed to be excreted. The GST actuates the organism’s detoxification of the toxic non-polar compounds in polar easy-excretion compounds by the insertion of the glutathione group in the molecule [59].

The lipid peroxidation was not observed in the nervous system, even by FOX or TBARS levels. Thus, it can be considered that the exposure to MEIP and Dfx brought benefits to the model organism, to the status of the defense system enzymatic antioxidants, and, therefore, proved to be good candidates for herb supplementation. In both of the performed tests, the aimed evaluation is the weak basis that is generated in cases of cell damage. Lipid peroxidation is considered to be the final result of the oxidative imbalance when the free radicals strongly react with the cell membranes, causing cellular, nuclear or molecular damage [60]. In this context, due to the habit of consuming herb mate, consolidated in the regional society and growing in the international market, the implementation of supplementation with Dfx can help reduce the oxidative damage in the body, preventing chronic diseases, thus being a good option for dieters.

Finally, *Ilex paraguariensis* is known due to its high antioxidant capacity as well as its anticancer [61] and anti-aging [62] effects. Heavy metals lead to an accelerated process of aging, due to the interaction with the nervous system affecting the damage progress as we previously demonstrated for cooper [63]. The addition of/supplementation with Dfx, to transform the *I. paraguariensis* extract into something that is safer and healthier, will increase the beneficial effects of this plant.

## 5. Conclusions

Here we show that adding Dfx improves the benefits of the extract, and reduces the toxicity of metals. So, the antioxidant potential of the extract could be used to prevent diseases and promote a healthy life. In addition, in zebrafish, an anxiolytic-like behavior was observed with increased intraspecific sociability, indicating positive points in the supplementation of the extract. In addition, microencapsulation can be considered efficient due to the morphological observation of the particles.

## Figures and Tables

**Table 1:** Statistical discrimination of the results.

Parameter	Figure		F Value	*p* Value
***Novel Tank Test***				
***Time spent in the top zone***	2A	Interaction	F_1,44_ = 2.325	0.1345
		Herb effect	F_1,44_ = 14.98	0.0004
		Dfx effect	F_1,44_ = 12.22	0.0011
***Time spent in the bottom zone***	2B	Interaction	F_1,44_ = 11.98	0.0012
		Herb effect	F_1,44_ = 2.528	0.1190
		Dfx effect	F_1,44_ = 38.38	<0.0001
***Latency to top***	2C	Interaction	F_1,44_ = 0.505	0.4811
		Herb effect	F_1,44_ = 8.444	0.0057
		Dfx effect	F_1,44_ = 7.353	0.0095
***Distance traveled***	2D	Interaction	F_1,44_ = 0.531	0.4699
		Herb effect	F_1,44_ = 10.94	0.0019
		Dfx effect	F_1,44_ = 0.523	0.4654
***Social Preference Test***				
***Time spent at the conspecific segment***	3A	Interaction	F_1,52_ = 0.7465	0.3915
		Herb effect	F_1,52_ = 15.26	0.0003
		Dfx effect	F_1,52_ = 15.05	0.0003
***Time far from the conspecific segment***	3B	Interaction	F_1,52_ = 0.7059	0.4047
		Herb effect	F_1,52_ = 4.714	0.0345
		Dfx effect	F_1,52_ = 14.37	0.0004
***Crossing number***	3C	Interaction	F_1,52_ = 13.45	0.0006
		Herb effect	F_1,52_ = 0.3301	0.5681
		Dfx effect	F_1,52_ = 6.344	0.0149
***Biochemical parameters***				
***ALAD activity***	4A	Interaction	F_1,32_ = 0.8297	0.3692
		Herb effect	F_1,32_ = 0.6972	0.4099
		Dfx effect	F_1,32_ = 1.475	0.2334
***AChE activity***	4B	Interaction	F_1,56_ = 0.1121	0.7390
		Herb effect	F_1,56_ = 3.756	0.0577
		Dfx effect	F_1,56_ = 11.25	0.0014
***Cortisol levels***	4C	Interaction	F_1,72_ = 16.19	0.0001
		Herb effect	F_1,72_ = 12.98	0.0006
		Dfx effect	F_1,72_ = 31.83	<0.0001
***SOD activity***	5A	Interaction	F_1,44_ = 17.08	0.0002
		Herb effect	F_1,44_ = 0.7868	0.3799
		Dfx effect	F_1,44_ = 5.858	0.0197
***CAT activity***	5B	Interaction	F_1,40_ = 41.80	<0.0001
		Herb effect	F_1,40_ = 2.382	0.1306
		Dfx effect	F_1,40_ = 5.554	0.0234
***GST activity***	5C	Interaction	F_1,60_ = 0.07510	0.7850
		Herb effect	F_1,60_ = 2.094	0.1531
		Dfx effect	F_1,60_ = 0.5466	0.4626
***FOX brain***		Interaction	F_1,36_ = 1.334	0.2557
		Herb effect	F_1,36_ = 0.8083	0.3746
		Dfx effect	F_1,36_ = 0.4564	0.5036
***TBARS brain***		Interaction	F_1,40_ = 0.5606	0.4584
		Herb effect	F_1,40_ = 1.809	0.1862
		Dfx effect	F_1,40_ = 2.259	0.1407
***TBARS whole body***		Interaction	F_1,4 0_ = 7.321	0.0100
		Herb effect	F_1,40_ = 135.6	<0.0001
		Dfx effect	F_1,40_ = 34.19	<0.0001

## Data Availability

The data that support the findings of this study are available from the corresponding author upon reasonable request.
